# Dicer Is Involved in Cytotoxicity and Motor Impairment Induced by TBPH Deficiency

**DOI:** 10.3390/cimb47060442

**Published:** 2025-06-10

**Authors:** Xiang Long, Yijie Wang, Hongrui Meng

**Affiliations:** Institute of Neuroscience, Soochow University, Suzhou 215123, China; dragonx0303@163.com (X.L.); yijiewang0624@163.com (Y.W.)

**Keywords:** amyotrophic lateral sclerosis, TBPH, *Drosophila*, Dicer

## Abstract

TDP-43 is an RNA-binding protein linked to amyotrophic lateral sclerosis (ALS), possibly associated with a role in miRNA biogenesis, which is still not fully understood. Herein we investigated the impact of the *Drosophila* homolog of TDP-43, TBPH, on genes related to miRNA biogenesis. A TBPH knockout significantly reduced mRNA transcription and protein levels of DCR-1 and DCR-2, whereas an overexpression of DCR-1 and DCR-2 in a TBPH knockdown background exacerbated compound eye damage, with variations in severity that were sex-dependent. Neuronal TBPH RNAi consistently shortened lifespan, with males and females exhibiting distinct survival profiles. DCR-1 and DCR-2 knockdown worsened the locomotor defects induced by TBPH deficiency, thus reinforcing the functional link between TBPH and DCR. In TBPH-deficient flies, the pharmacological activation of Dicer promoted reverse locomotion behavior, with a preference for backward movement. Overall, we show that TBPH is a key regulator of DCR protein expression, highlighting its conserved role in miRNA dysregulation associated with motor function and cytotoxicity in ALS-like pathology in *Drosophila* models.

## 1. Introduction

Amyotrophic lateral sclerosis (ALS) is a rapidly progressing disease that causes the loss of motor neurons in the brain and spinal cord, leading to skeletal muscle weakness and wasting. The presence of cytoplasmic inclusions of TAR DNA-binding protein-43 (TDP-43) in affected neurons is a major pathological feature and is observed in approximately 95% of ALS patients [1]. In pathological conditions, the heterogeneous nuclear ribonucleoprotein shuttles between the nucleus and cytoplasm and forms detergent-resistant aggregates commonly observed in both ALS and frontotemporal dementia (FTD) [2,3]. TDP-43 is involved in RNA metabolism, such as in alternative splicing, transcriptional regulation, transport, mRNA stability, and translation [4]. The disruption of the mechanism that regulates its nuclear–cytoplasmic exchange has been linked to neurodegeneration [3]. TDP-43 is involved in irregular RNA metabolism and altered neuronal transcript splicing, including dysregulation of non-coding RNAs. These disruptions lead to aberrant cellular pathways and suggest multiple potential disease mechanisms [5,6,7].

*Drosophila* is an established model for investigating TDP-43 in ALS and other neurodegenerative disorders [8,9]. Indeed, in this model, the loss of its TDP-43 homolog (TBPH) results in an ALS-like phenotype, which includes motor defects, toxicity, and impaired locomotor activity [10,11]. Previously, we systematically screened for the genetic interaction of neurodegenerative fly phenotypes of mutant *CHCHD2*, a gene related to familial Parkinson’s disease that is linked to the misexpression and redistribution of endogenous TBPH [12]. Other studies have shown that TDP-43 can affect miRNA processing through its interaction with protein complexes containing Drosha and Dicer [13]. TDP-43, along with other disease-related RNA-binding proteins such as fused in sarcoma (FUS), promotes gene silencing by interacting with miRNA and mRNA targets, a mechanism that may contribute to degeneration in ALS/FTD [14,15]. However, none of the available models have yet identified the genetic and physical interactions underlying degenerative phenotypes between TDP-43 and molecules responsible for miRNA processing.

Mature miRNAs originate in the nucleus from endogenous hairpin-shaped transcripts in the nucleus and are then transported to the cytoplasm [16,17]. Nuclear RNase III, Drosha, combines with its essential cofactor DGCR8 (also known as Pasha in *Drosophila*) and cuts the stem loop to release pre-miRNA, thus initiating maturation after transcription [18,19,20]. In the cytoplasm, the pre-miRNA undergoes cleavage by Dicer to yield a mature miRNA of approximately 22 nucleotides. In *D. melanogaster*, DCR-1 and DCR-2 are essential for the biogenesis of endogenous miRNAs and the production of exogenous siRNAs, despite each enzyme possessing distinct RNase III activity [21]. DCR-1 interacts with Loquacious (Loqs) during the processing of double-stranded RNA, resulting in frequent internal mismatched miRNA-miRNA* duplexes [22,23,24]. Dicer coordinates with the DCR-2/R2D2 complex, forms an RNA-induced silencing complex (RISC), binds to a perfectly complementary miRNA, and is sorted into Argonaute (AGO) proteins [25,26]. Multiple biological pathways are regulated by miRNA, which can target one or several genes after transcription in both mammals and fruit flies.

In the present study, we employed TBPH-knockdown and -knockout *Drosophila* models to investigate the interaction of TDP-43 (TBPH) with molecules involved in miRNA biogenesis. We demonstrate that TBPH deficiency downregulates both the transcription and translation of Dicer, the key enzyme in miRNA processing. The compound eye toxicity and locomotor defects caused by the loss of TBPH function are significantly modulated by altering Dicer expression. These findings suggest that Dicer contributes to the ALS-like degenerative phenotypes induced by TBPH (dTDP-43) deficiency in *Drosophila* models and offer valuable insights into the role of miRNAs in the pathogenesis of ALS.

## 2. Materials and Methods

### 2.1. Drosophila Strains and Husbandry

Fly stocks were maintained at 25 °C under a 12 h light/12 h dark cycle and fed a standard diet of cornmeal, yeast, and agar. For enoxacin treatment, parental crosses were established on standard cornmeal medium supplemented with either the drug or vehicle until progeny larvae were subjected to the crawling assay. The RNAi lines used in this study were sourced from the Vienna Drosophila Resource Center (VDRC) and the Bloomington Drosophila Stock Center (BDSC). The RNAi constructs in these fly lines targeted specific regions of *Drosophila* TBPH, corresponding to residues 81–181 (UAS-TBPH-IR81-181; VDRC number 38377) and 564–570 (UAS-TBPH-IR564-570; BDSC number 39014). These lines express TBPH double-stranded RNA (dsRNA; inverted repeats, IRs) directed against distinct regions of TBPH mRNA. To achieve the tissue-specific expression of TBPH dsRNA, flies with the UAS-TBPH-IR genotype were crossed with flies carrying GMR-GAL4, Elav-GAL4, or D42-GAL4 drivers, enabling targeted expression in the compound eyes, pan-neuronal cells, or motor neurons, respectively. The *Drosophila* strains used in this study are detailed in the Table S2. Unless otherwise specified, male flies were used for all experiments.

### 2.2. Locomotion Tracking Behavior and Larval Crawling Assay

Adult fly and larval locomotor assays were performed as previously described [27], with minor modifications. Adult males were rapidly transferred to a 5 cm diameter open-field arena containing silica gel (leaving a 5 mm gap from the lid). Video recordings captured the free movement of adult flies or third-instar larvae for 1 min using a high-resolution digital camera (see Table S2). Open-source Fiji (ImageJ) software version 1.53v, combined with an animal tracker plugin [28], was used to monitor and analyze movement patterns in the videos. Locomotion parameters (distance, immobility time, and mean velocity) were extracted and converted into digital format. The deflection angle of *Drosophila* larvae between two consecutive frames was calculated using the cosine theorem based on three continuous coordinates from the track file. The cosine value (X) of the angle was determined as follows: (SQRT ((Cn-Cn-1) ^ 2 + (Dn-Dn-1) ^ 2)) ^ 2 + (SQRT ((Cn-1-Cn-2) ^ 2 + (Dn-1-Dn-2) ^ 2)) ^ 2-(SQRT ((Cn-Cn-2) ^ 2 + (Dn-Dn-2) ^ 2)) ^ 2/(2 × SQRT ((Cn-1-Cn-2) ^ 2 + (Dn-1-Dn-2) ^ 2)) × (SQRT ((Cn-Cn-1) ^ 2 + (Dn-Dn-1) ^ 2))”. The function “ACOS (X) × 180/PI ()” was used to calculate the deflection angle. Larval movement was categorized into “Stillness” and “Motion”. Stillness included two states: “Pause” (deflection angle <20° or >160°) and “Head sweep” (deflection angle between 20° and 160°). Motion included “Forward” (deflection angle < 20°), “Turn” (deflection angle between 20° and 160°), and “Reverse” (deflection angle > 160°).

### 2.3. Eye Imaging

Adult fly eyes were imaged using an Olympus SZX7 microscope (Olympus, Tokyo, Japan) equipped with a SITUOLI STL-P830EISPM3C CMOS camera. Individual images were processed with Adobe Photoshop CS6 (Adobe, San Jose, CA, USA).

### 2.4. RNA Extraction and RT-qPCR

Total RNA was extracted using Trizol (Invitrogen, Carlsbad, CA, USA) from larvae, or adults, following the manufacturer’s instructions, including a DNase treatment. cDNA was synthesized from 500 ng of total RNA using the HiScript^®^ III RT SuperMix kit (Vazyme, Nanjing, China). Real-time PCR was performed using ChamQ Universal SYBR qPCR Master Mix (Vazyme), and the primers used are listed in Table S1. Relative expression (fold change) was calculated using the ΔΔCT method, with rp49 serving as the normalization control.

### 2.5. Western Blot

The whole lysate of adult flies or larvae abdominal was mixed with the SDS sample loading buffer (200 mM Tris-HCl pH 6.8, 8% (*w*/*v*) SDS, 0.4% (*w*/*v*) bromophenol blue, 40% (*v*/*v*) glycerol, 4% (*v*/*v*) 2-mercaptoethanol) and heated at 95 °C for 5 min. The samples were subjected to SDS-PAGE electrophoresis, and proteins were transferred to a 0.45 μm PVDF membrane (Merck Millipore, Burlington, VT, USA) via wet or semi-dry transfer. Nonspecific binding was blocked with 5% non-fat skim milk or BSA in TBST for 30 min. Primary antibodies were diluted and incubated overnight at 4 °C, followed by incubation with HRP-conjugated secondary antibodies at room temperature for 2 h, depending on the primary antibody. Signals were visualized using ECL Luminata™ Western HRP Substrates (Merck Millipore) and imaged using the ChemiDoc system (Bio-Rad, Hercules, CA, USA). Exposure times were adjusted for each blot based on individual protein expression levels to ensure robust quantification. Primary and secondary antibodies used in this study are listed in the Table S2.

### 2.6. Lifespan Analysis

Progeny flies of the corresponding genotypes were collected and separated by sex. For each genotype, at least 120 flies were used. The flies were distributed into vials at a density of 20 flies per vial. All flies were maintained under controlled conditions (25 °C, 60% humidity, 12 h light/12 h dark cycle). Every two days, the flies were transferred to fresh cornmeal food vials without anesthesia. The number of dead flies was recorded every 12 h until all individuals had died. Survival data were recorded using Microsoft Excel (Office 2019) and subsequently analyzed using GraphPad Prism 9.5 to generate survival curves and calculate mean and median lifespans.

### 2.7. Acquisition of Subcellular Localization Images

Adult fly brains were dissected and fixed with pre-cooled 4% paraformaldehyde (PFA). The samples were washed three times with PBST (0.05% Tween-20 in PBS) for 10 min each. The brains were then blocked in 10% goat serum for 30 min at room temperature. Primary antibodies were applied and incubated overnight at 4 °C in the dark. Following three additional washes with PBST, secondary antibodies were applied and incubated for 2 h at room temperature. The brains were subsequently mounted on glass slides using an anti-fade mounting medium. Images were acquired using a Zeiss LSM700 confocal microscope (Zeiss AG, Oberkochen, Germany) equipped with a 63× oil immersion objective and analyzed with Fiji (ImageJ) software version 1.53v.

### 2.8. Statistical Analysis

Statistical analyses were performed using GraphPad Prism version 9.5 (GraphPad Software, Boston, MA, USA). For comparisons between two groups, a two-tailed unpaired Student’s *t*-test was used. For multiple group comparisons, one-way ANOVA followed by Tukey’s post hoc test or two-way ANOVA followed by Bonferroni’s post hoc test was performed. When the assumptions of normality or homogeneity of variance were not met, Welch’s ANOVA or non-parametric tests were employed, followed by Dunnett’s or Dunn’s post hoc test, as appropriate. The data are presented as the mean ± standard error of the mean (SEM). For lifespan experiments, statistical analysis of all fly lifespans was conducted using the non-parametric Kruskal–Wallis test, followed by Dunn’s method for post hoc comparisons. Statistical significance was defined as * *p* < 0.05, with ** *p* < 0.01, *** *p* < 0.001, and **** *p* < 0.0001.

## 3. Results

### 3.1. The Regulatory Role of TBPH on DCR Proteins

**Figure 1:**
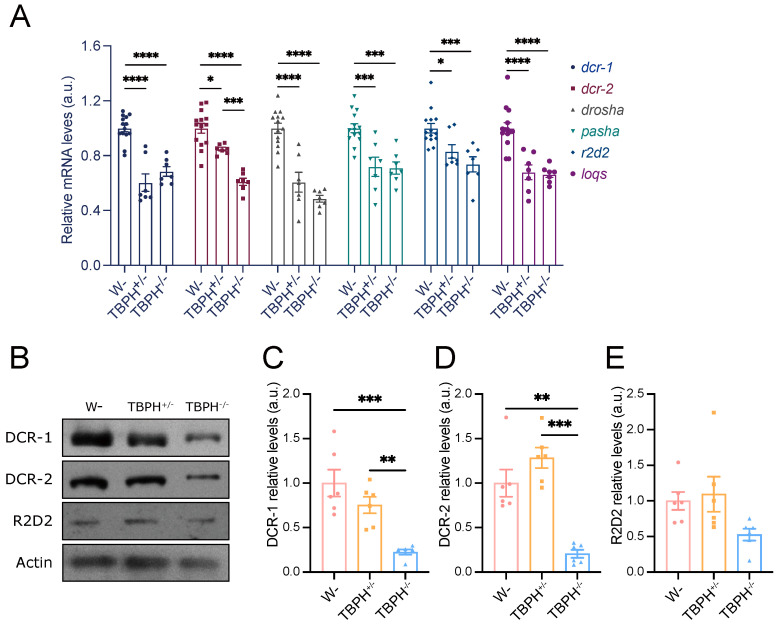
Regulatory role of TBPH in genes associated with miRNA biogenesis. (**A**) RT-qPCR analysis of the mRNA levels of *dcr-1*, *dcr-2*, *drosha*, *pasha*, *r2d2*, and *loqs* in TBPH deficiency flies. Expression of *rp49* mRNA was used as endogenous control; (**B**–**E**) Western blot analyses of the effect of DCR-1 (**C**), DCR-2 (**D**), and R2D2 (**E**) in TBPH^+/−^ and TBPH^−/−^ flies and the relative protein expression were quantified (*n* = 6 flies for each genotype). Data were analyzed using one-way ANOVA with Tukey’s post hoc test. (* *p* < 0.05, ** *p* < 0.01, *** *p* < 0.001, **** *p* < 0.0001). Error bars represent mean ± SEM.

To investigate whether TBPH regulates miRNA biogenesis-related genes, we first examined the mRNA and protein levels of these genes in TBPH knockout (TBPH^Δ23^) and wild-type (W^1118^) Drosophila larvae. Compared to wild-type larvae, both TBPH^+/−^ and TBPH^−/−^ genotypes exhibited significant reductions in mRNA levels of *dcr-1*, *dcr-2*, *drosha*, *pasha*, *r2d2*, and *loqs* (Figure 1A). Notably, the mRNA level of *dcr-2* was significantly different between TBPH^+/−^ and TBPH^−/−^ larvae. Consistent with mRNA findings, DCR-1 and DCR-2 protein levels were significantly reduced in TBPH^−/−^ larvae (Figure 1B). However, TBPH^+/−^ larvae did not display a significant reduction in protein levels (Figure 1C,D). R2D2 protein expression was at similar levels across the three genotypes (Figure 1E). Overall, these results demonstrate that TBPH plays a critical regulatory role in the expression of miRNA biogenesis-related proteins.

### 3.2. Effects of DCR on TBPH-Induced Compound Eye Phenotypes

**Figure 2:**
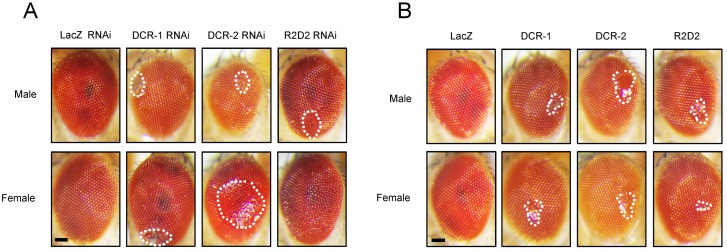
Effect of miRNA biogenesis-related genes on fly compound eye damage. (**A**) The GMR-GAL4 driver was used to express UAS-DCR-1 RNAi, UAS-DCR-2 RNAi, and UAS-R2D2 RNAi in the compound eyes of flies. Representative images illustrating compound eye damage are presented, with the upper panel showing male fly compound eyes and the lower panel showing female fly compound eyes. All images were cropped to uniform dimensions, and areas of compound eye damage are indicated by white dashed lines. (**B**) GMR-GAL4 was used to drive the expression of UAS-DCR-1, UAS-DCR-2, and UAS-R2D2 in the compound eyes of flies. Representative images of eye damage are shown, with the upper panel displaying male flies and the lower panel displaying female flies. All images were cropped to the same dimensions, and the damaged eye regions are indicated by white dashed lines. Scale bar: 50 μm.

We then explored the relationship between *Drosophila* phenotypes and the regulation of miRNA biogenesis-related genes by TBPH. We utilized the GMR-GAL4 driver to induce either the overexpression or knockdown of key miRNA biogenesis pathway components (i.e., DCR-1, DCR-2, and R2D2) in the compound eyes of flies. Compared to the control group, both the overexpression (Figure 2A) and knockdown (Figure 2B) of these components resulted in observable damage to phenotypes in the compound eyes, in both males and females, although to different extents. This suggests a significant regulatory role for miRNA biogenesis-related genes in the compound eye phenotype.

**Figure 3:**
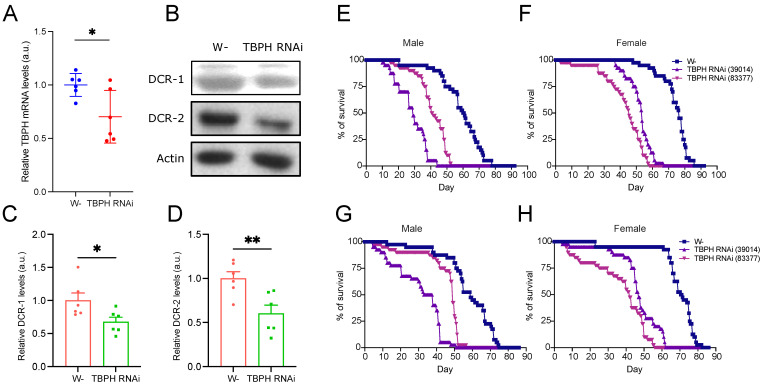
TBPH RNAi reduces DCR expression and shortens lifespan. (**A**) qPCR analysis of TBPH mRNA levels in the thorax of wild-type and whole-body TBPH knockdown flies (*n* = 6); (**B**) representative Western blot images showing DCR-1 and DCR-2 protein expression levels following TBPH knockdown, with actin as the loading control; (**C**,**D**) quantification of DCR-1 (**C**) and DCR-2 (**D**) protein expression levels from (**B**) (*n* = 6); (**E**,**F**) lifespan analysis of male (**E**) or female (**F**) flies with pan-neuronal (elav-GAL4>) TBPH knockdown; (**G**,**H**) lifespan analysis of male (**G**) and female (**H**) flies with motor neuron-specific (D42-GAL4>) TBPH knockdown. Data are shown as mean ± SEM and were analyzed using a two-tailed *t*-test. * *p* < 0.05, ** *p* < 0.01.

To assess the influence of sex, we used the da-GAL4 driver to ubiquitously knock down TBPH (TBPH RNAi #38377) and evaluated DCR protein expression changes in the thorax. qPCR confirmed a ~30% TBPH knockdown efficiency (Figure 3A), whereas reduction in protein levels was observed for both DCR-1 (>30%) and DCR-2 (>40%) (Figure 3B–D), consistent with prior WB results from TBPH knockout flies.

To validate lifespan effects, we performed TBPH knockdown in pan-neuronal and motor neuron-specific contexts using two independent TBPH RNAi strains (TBPH RNAi #38377 and #39014). Both strains significantly reduced fly lifespan observed in wild-type males and females (Figure 3E,F and Table S3). For TBPH RNAi (#38377) and TBPH RNAi (#39014), mean and median lifespans were about 40 and 41 days, and 27 and 26 days, respectively (compared to 58 and 60 days for wild-type males). In females, mean and median lifespans for TBPH RNAi (#38377) and TBPH RNAi (#39014) were approximately 43 and 46 days, and 51 and 53 days, respectively (compared to 72 and 74 days for the wild type). Similarly, motor neuron-specific TBPH knockdown confirmed lifespan reduction (Figure 3G,H and Table S4). In males, mean and median lifespans for TBPH RNAi (#38377) and TBPH RNAi (#39014) were approximately 44 and 48 days and 27 and 31 days (compared to 53 and 55 days for the wild type). In females, mean and median lifespans for TBPH RNAi (#38377) and TBPH RNAi (#39014) were about 37 and 42 days and 48 and 47 days (compared to 67 and 70 days for the wild type). These consistent results demonstrate that TBPH knockdown reduces lifespan, underscoring the conserved role of TDP-43 in this model system. The significant lifespan variations between strains (#38377 and #39014) and sexes may be linked to differences in the mRNA regions targeted by the dsRNA expressed in these strains.

**Figure 4:**
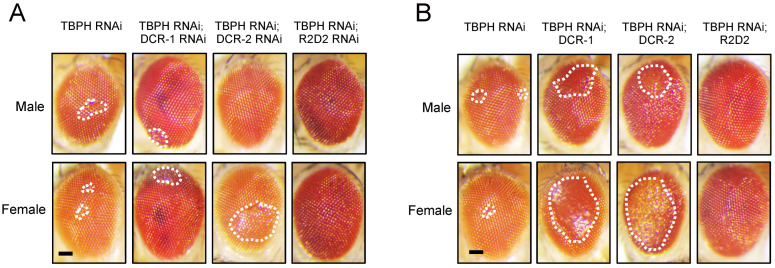
Modulating DCR expression impact on TBPH RNAi-induced eye damage. (**A**) GMR-GAL4 was used to drive the expression of UAS-TBPH RNAi in *Drosophila* compound eyes. Additionally, GMR-GAL4 was used to co-express UAS-TBPH RNAi with UAS-DCR-1 RNAi, UAS-DCR-2 RNAi, and UAS-R2D2 RNAi; (**B**) GMR-GAL4 was used to co-express UAS-TBPH RNAi with UAS-lacZ, UAS-DCR-1, UAS-DCR-2, and UAS-R2D2. Representative images of eye damage are shown. All images were cropped to the same dimensions, and white dashed lines indicate the damaged eye regions. Scale bar: 50 μm.

To further investigate the role of TBPH in miRNA biogenesis, we generated *Drosophila* strains where miRNA biogenesis-related genes were overexpressed (Figure 4B) or knocked down (Figure 4A) in a TBPH RNAi background (TBPH RNAi #38377). Using the GMR-GAL4 driver to express these genes in compound eyes, we observed that TBPH RNAi induced phenotypes similar to those caused by the knockdown of miRNA biogenesis-related genes, resulting in comparable eye damage. However, the overexpression of DCR-1 and DCR-2 significantly worsened the compound eye damage caused by TBPH knockdown, whereas the RNAi-mediated knockdown of the DCR gene did not notably alter the TBPH knockdown-induced phenotype. These findings suggest a potential functional interplay between TBPH and miRNA biogenesis-related genes.

### 3.3. Potential Direct Interaction Between TBPH and DCR Proteins

**Figure 5:**
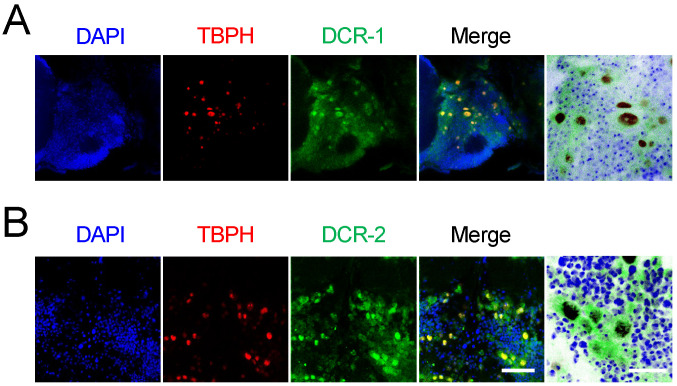
Colocalization of TBPH and endogenous Dicer in motor neurons. (**A**,**B**) Subcellular localization of TBPH-tagged RFP (red) in motor neurons (D42-Gal4>) with DCR-1 (**A**) and DCR-2 (green) (**B**) of 5-day-old flies. Merged images and magnified overlays illustrate the subcellular colocalization of TBPH and DCR proteins (brown) in motor neurons. Scale bars: 25 μm (left) and 10 μm (right magnified). Nuclei were counterstained with DAPI (blue) in merged images.

To further explore the observed relationship between TBPH and DCR, we expressed TBPH tagged with red fluorescent protein (RFP) in adult motor neurons while labeling DCR proteins with anti-DCR antibodies (green fluorescence). Confocal microscopy analysis of fly brains revealed that both DCR-1 (Figure 5A) and DCR-2 (Figure 5B) co-localized with TBPH at the subcellular level, suggesting potential direct interaction.

### 3.4. DCR Knockdown Exacerbates Motor Defects Induced by TBPH Knockdown

**Figure 6:**
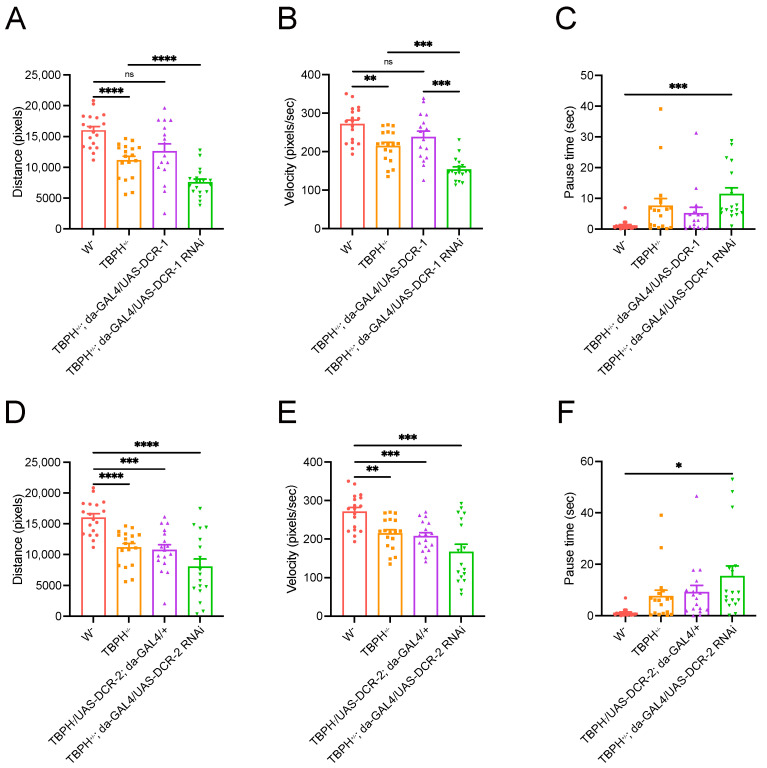
DCR RNAi exacerbates locomotor defects induced by TBPH RNAi. (**A**–**C**) Locomotion distance (**A**), velocity (**B**), and pause time (**C**) in the spontaneous movement assay of flies expressing or knocking down DCR-1 in the TBPH^+/−^ genetic background; (**D**–**F**) locomotion distance (**D**), velocity (**E**) and pause time (**F**) in the spontaneous movement assay of flies expressing or knocking down DCR-2 in the TBPH^+/−^ genetic background. Data are shown as mean ± SEM and were analyzed using a Welch ANOVA with Dunnett multiple comparison tests. * *p* < 0.05, ** *p* < 0.01, *** *p* < 0.001, **** *p* < 0.0001, ns *p* > 0.05.

We have established that TBPH knockdown significantly reduces lifespan in *Drosophila*, producing a pathological phenotype reminiscent of ALS. Also, we have observed a substantial regulatory effect of TBPH on DCR protein expression. However, it remains unclear whether the TBPH regulation of DCR proteins is associated with motor impairment in flies. To address this, we conducted locomotor analysis on heterozygous adult flies of TBPH KO, as the homozygotes are lethal [10]. Compared to wild-type flies, flies with a TBPH^+/−^ genetic background exhibited a significant reduction in both movement distance and speed, and also an increase in immobility time (Figure 6). However, the overexpression of DCR-1 in that background led to restored locomotor ability to wild-type levels (Figure 6A,B). In contrast, knocking down DCR-1 in the same background further reduced movement distance and speed while increasing immobility time (Figure 6A–C). Similarly, although DCR-2 overexpression did not significantly improve locomotor ability in TBPH^+/−^ flies (Figure 6D–F), its knockdown markedly increased immobility time (Figure 6F). These findings collectively demonstrate that the knockdown of DCR genes exacerbates the motor defects induced by TBPH knockdown.

### 3.5. Active DCR Affects Locomotor Deficiency Induced by TBPH KO

**Figure 7:**
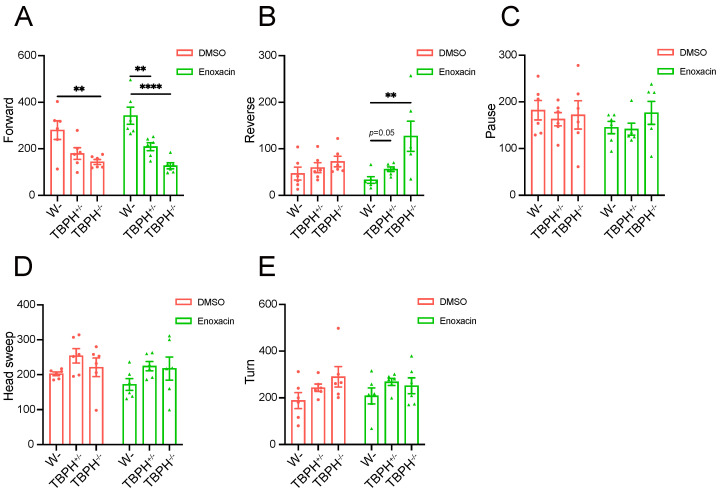
Active DCR affects TBPH KO-induced locomotor deficiency. (**A**–**E**) Locomotor analysis of wild-type, TBPH^+/−^, and TBPH^−/−^ larvae following DMSO or enoxacin treatment, including Forward (**A**), Reverse (**B**), Pause (**C**), Head sweep (**D**), and Turn (**E**). Data are shown as mean ± SEM and were analyzed using a one-way ANOVA with Tukey’s multiple comparisons test and a two-way ANOVA with Bonferroni’s multiple comparisons test. ** *p* < 0.01, **** *p* < 0.0001.

To further substantiate that TBPH influences *Drosophila* locomotor ability by regulating DCR, we treated larvae with enoxacin, a compound previously reported to enhance miRNA and siRNA biogenesis by facilitating Dicer recruitment to the AGO2 complex [29]. We analyzed larval locomotor behavior by categorizing movement states within a 1 min period into “Stillness” and “Motion”, as in the previous report [30]. Stillness was subdivided into “Pause” and “Head sweep”, while motion encompassed “Forward”, “Reverse”, and “Turn”. In flies treated with DMSO, a deficiency of TBPH induced alterations in locomotor states, predominantly in the “Forward” category. Compared to wild-type larvae, complete TBPH knockout significantly reduced the frequency of the “Forward” movement (Figure 7A). Although in TBPH-knockout flies, changes were observed in other movement categories, these did not reach statistical significance (Figure 7B–E). However, following enoxacin treatment, the locomotor tracking activity in TBPH-knockout larvae was modestly improved (Table S5). Compared to the control group, TBPH-knockout larvae treated with enoxacin exhibited an increase in the “Reverse” movement state (Figure 7B). Conversely, TBPH^+/−^ and TBPH^−/−^ still showed a significant reduction in the “Forward” movement state despite enoxacin treatment (Figure 7A). These results provide further evidence of a close relationship between TBPH and DCR in regulating locomotor behavior in *Drosophila*.

## 4. Discussion

Despite the known role of ALS-associated protein TDP-43 in RNA metabolism, the physio-pathological roles of miRNA processing that are involved in neurodegeneration remain incompletely understood. Using a *Drosophila* null mutant model, we show that the transcriptional machinery of miRNA biogenesis was reduced in the heterozygote and homozygote TBPH-null flies. Additionally, we showed that endogenous DCR-1 and DCR-2, two principal components of the small RNA processing pathway, are downregulated in the absence of endogenous TBPH. This suggests that defects in the activity of this endoribonuclease might be responsible for post-transcriptionally silencing target genes. Parallel studies have reported that the knockdown of TDP-43 in human neuroblastoma cell lines decreases Dicer and TBPH physically interact with DCR-2 mRNA and its protein product [31], suggesting that TDP-43/TBPH is essential for the transport of the RISC inside or outside the nucleus or may contributes to the binding and recognition of RNA targets. In agreement with this, we found that DCR-1 is also reduced, indicating that TBPH is involved in the processing of endogenous miRNA in vivo.

Dicer, a multidomain endonuclease, belongs to the fourth class of type III ribonuclease (RNase III) enzymes and is conserved in humans and *Drosophila melanogaster* [32]. DCR-1 functions enzymatically by working together with Loqs to convert pre-miRNA into a miRNA/miRNA* duplex [33]. A deficiency of DCR-1 in rodent motor neurons results in symptoms resembling spinal muscular atrophy which contributes to β-amyloid accumulation and dopamine loss [34,35]. Notably, the knockdown or expression of DCR-1, DCR-2, and R2D2 causes a morphological defect in the rough compound eye phenotype of adults, which is a useful visible marker for evaluating degeneration caused by specific genes and genetic interactions with target genes [36]. A genetic interaction study of TBPH RNAi and *Dicer* and other genes showed that both DCR-1 and DCR-2 expression exacerbate TBPH RNAi-induced rough eye phenotypes, whereas knockdowns induce minor effects. This indicates that Dicer expression may be part of a functional negative feedback loop triggered by the reduced TBPH levels in cytoplasmic and nuclear processes [37]. However, additional evidence is needed to confirm this hypothesis.

In our results, sex exerts a notable influence on both compound eye phenotype and lifespan in flies. These are modulated by TBPH, which regulates genes related to miRNA biogenesis, particularly DCR proteins. In experiments using the GMR-GAL4 driver, the overexpression or knockdown of DCR-1, DCR-2, and R2D2 in the compound eyes resulted in observable rough eye phenotypes, with effects consistent across males and females. However, the severity of these phenotypes, when caused by TBPH RNAi combined with Dicer-manipulated expression, is more severe in females, a finding consistent with our previous observation [12]. This aligns with TBPH RNAi exacerbating eye damage when combined with DCR-1 and DCR-2 overexpression, indicating a functional interplay between TBPH and DCR proteins. This may be sex-dependent, suggesting sex-specific differences in sensitivity or different compensatory mechanisms.

Lifespan analysis further underscores sex-dependent outcomes. We used two RNAi fly lines that targeted the region corresponding to residues 81–181 and 517–531 to examine the lifespan of flies in both males and females [38]. When crossed with pan-neuronal or motoneuron-specific lines, the TBPH knockdown targeting residues 517–531 had a more severely shortened lifespan in males, consistent with a previous report [38], and all the flies showed a rough eye phenotype. However, female flies with the same background showed a significantly shorter lifespan in the lines of TBPH knockdown targeting residues 81–181 (Tables S3 and S4). Although the precise mechanism is not known, we note that a prospective cohort study revealed an elevated risk for ALS and a worsened prognosis, specifically in female smokers [39]. Given that TBPH deficiency results in rough compound eye and longevity assays with sex-dependent trends, exploring molecular and genetic components that impact sex-related differences in these models may explain the observed phenotype disparities in males and females. These components may be sex-specific cellular mechanisms, genetic modifiers, and hormone regulation.

Finally, in addition to the reduction in Dicer mediated by TBPH deficiency, we found that knockdown Dicer, especially DCR-1, exacerbates the locomotor defects induced by TBPH deficiency. However, the overexpression of DCR-1 and DCR-2 did not fully rescue these locomotor defects. A pharmacological treatment involving enoxacin, a compound that activates the Dicer protein in mammalian cell lines, effectively promoted reverse locomotion behavior in TBPH-deficient flies.

TDP-43 carries two RNA recognition motifs and affects the expression of certain miRNAs and binding affinity to distinct miRNAs, thus controlling miRNA processing and function in humans [40,41], *Drosophila* [15,42], and mammalian cells [13,43]. Although the exact nature of these interactions is unclear, we show that TBPH is required for the intracellular localization of the RISC and/or its transport inside or outside the nucleus, as well as the recognition of RNA targets by affecting endogenous miRNA maturation. Based on these findings, we suggest a model in which the excessive abnormal miRNA maturation caused by TBPH deficiency is linked to Dicer, resulting in targeted gene expression that may cause cytotoxicity and behavioral abnormalities.

In summary, our study reveals that TBPH, the *Drosophila* homolog of the ALS-associated protein TDP-43, plays a crucial role in the regulation of the biogenesis of miRNA expression. TBPH depletion leads to significant alterations in the expression of key miRNA biogenesis proteins, such as DCR-1 and DCR-2. Importantly, Dicer expression significantly dysregulated in TBPH-deficient flies. Its restoration alleviates motor deficits, suggesting a critical role in neurodegeneration. These findings highlight the conserved regulatory role of TBPH in miRNA processing through interaction with Dicer and its potential involvement in ALS pathogenesis.

## 5. Conclusions

Our results suggest that miRNA biogenesis-related genes are controlled by TBPH, and Dicer manipulation (expression or activation) is linked to locomotor impairment and compound eye phenotypes. Our study demonstrates that miRNA biogenesis-related genes affect the cytotoxicity and behavioral defects triggered by TBPH loss, thus highlighting the effects of miRNA processing in ALS/FTD pathology.

## Data Availability

The data are contained within the article.

## Supplementary Materials

The following supporting information can be downloaded at https://www.mdpi.com/article/10.3390/cimb47060442/s1.

## Abbreviations

The following abbreviations are used in this manuscript:

TDP-43	DNA/RNA-binding protein 43
TBPH	DNA/RNA-binding protein 43 homolog
ALS	Amyotrophic lateral sclerosis
FTD	Frontotemporal dementia
miRNA	MicroRNA
AGO	Argonaute
Loqs	Loquacious
RISC	RNA-induced silencing complex
dsRNA	Double-stranded RNA
