# Supplementary material for: Dicer Is Involved in Cytotoxicity and Motor Impairment Induced by TBPH Deficiency
Source: Curr Issues Mol Biol. 2025 Jun 10;47(6):442. doi: 10.3390/cimb47060442 (PMC12192051; doi:10.3390/cimb47060442)
Supplement: Supplementary file 1 [file cimb-47-00442-s001.zip › cimb-3620640-supplementary.pdf]

## Supplemental data

### Table S1. Primer design for RT-qPCR to detect mRNA in this study

| Oligonucleotide sequences (Forward primer on top; Reverse primer below) | Oligo Identifier |
|---|---|
| GTCAGGCGATACATACAAAGGAAAAGAA<br>CGGTGCGAAATAGGAATCAGGTAA | *TBPH* |
| TCGAACTGATCGTGCTGGAAG<br>CAGTTGCTGCAGCTCACATCC | *DCR-1* |
| CGTGGCTTTAAGCTCCCTGA<br>ATTAACACCCCGGTGAGACC | *DCR-2* |
| AGAGTTCTTGGGCGACACA<br>TTGGGATTGGCATACACG | *drosha* |
| TGATGGTGACGGCGAAGAATA<br>ATCCCTCGGGTAGGACTTCAA | *pasha* |
| GGCGGATCGGGCTTACAAG<br>CGTTTCGCTGACGAACTTTAAGG | *loqs* |
| CGGAGGGTACGTCTGTAAAGT<br>ACAGTCGAATCCTTCATCAAGC | *R2D2* |
| AGCATACAGGCCCAAGATCG<br>TGTTGTCGATACCCTTGGGC | *rp49* |

**Table S2. Reagents and Tools Table**

| Reagent/Resource | Reference or | Identifier or Catalog Number |
|---|---|---|
| Antibodies | | |
| Rabbit anti-DCR-2 | Abcam | Cat #ab4732; RRID:AB_449344 |
| Rat anti-actin (W16197A) | Biolegend | Cat #664802; RRID: AB_2721349 |
| Peroxidase AffiniPure Goat Anti-Mouse IgG (H+L) | Jackson ImmunoResearch | Cat #115-035-003; RRID: AB_10015289 |
| Peroxidase AffiniPure Goat Anti-Rabbit IgG (H+L) | Jackson ImmunoResearch | Cat #111-035-003; RRID: AB_2313567 |
| HRP conjugated anti Rat IgG | Beyotime Biotechnology | Cat #A0192; RRID:AB_293901 |
| Rabbit anti-DCR-1 | in this study | N/A |
| Rabbit anti-R2D2 | in this study | N/A |
| Goat anti-mouse IgG (H+L) Alexa Fluor 488 | Invitrogen | Cat #A-11017; RRID:AB_2534084 |
| Chemicals, Enzymes, and other reagents | | |
| ChamQ Universal SYBR qPCR Master Mix | Vazyme | Cat #Q711 |
| ChamQ SYBR qPCR Master Mix | Vazyme | Cat #Q311 |
| HiScript III RT SuperMix for qPCR | Vazyme | Cat #R323 |

| | | |
|---|---|---|
| HiScript II Q Select RT SuperMix for qPCR | Vazyme | Cat #R233 |
| HiScript III 1st Strand cDNA Synthesis Kit | Vazyme | Cat #R312 |
| Chemicals, peptides, and recombinant proteins | | |
| Enoxacin | TargetMol Chemicals Inc | Cat# T0717L |
| Experimental models: Organisms/strains | | |
| *D. melanogaster: $W^{1118}$* | BDSC | RRID: BDRC_5905 |
| *D. melanogaster: UAS-DCR1* | BDSC | RRID: BDRC_36510 |
| *D. melanogaster: UAS-DCR1* | BDSC | RRID: BDRC_78265 |
| *D. melanogaster: UAS-DCR-1 RNAi* | BDSC | RRID: BDRC_24667 |
| *D. melanogaster: UAS-DCR-1 RNAi* | BDSC | RRID: BDRC_24666 |
| *D. melanogaster: UAS-DCR-2* | BDSC | RRID: BDRC_24650 |
| *D. melanogaster: UAS-DCR-2 RNAi* | BDSC | RRID: BDRC_60009 |
| *D. melanogaster: UAS-DCR-2 RNAi* | BDSC | RRID: BDRC_60008 |
| *D. melanogaster: UAS-R2D2* | BDSC | RRID: BDRC_14997 |
| *D. melanogaster: UAS-R2D2 RNAi* | BDSC | RRID: BDRC_26727 |
| *D. melanogaster: UAS-lacZ* | BDSC | RRID: BDRC_8529 |

| | | |
|---|---|---|
| *D. melanogaster: UAS-lacZ RNAi* | Gift from the Imai Lab | N/A |
| *D. melanogaster: UAS-TBPH RNAi* | VDRC | RRID: VDRC_38377 |
| *D. melanogaster: UAS-TBPH RNAi* | BDSC | RRID: BDRC_39014 |
| *D. melanogaster: Elav-Gal4* | BDSC | RRID: BDRC_8760 |
| *D. melanogaster: Gmr-Gal4* | BDSC | RRID: BDRC_1104 |
| *D. melanogaster: Gmr-Gal4* | SIBCB | RRID: BCF_483 |
| *D. melanogaster: D42-Gal4/TM6B* | SIBCB | RRID: BCF_114 |
| *D. melanogaster: UAS-TBPH RFP #8* | Gift from the Imai Lab | N/A |
| *D. melanogaster: TBPHΔ23/CyO,GFP* | Gift from the Feiguin Lab | N/A |
| Software and algorithms | | |
| Fiji | Fiji team | N/A |
| Animal Tracker Plugin | MTA-ELTE-NAPB Neuronal Cell Biology Research Group | N/A |
| Format factory software | PCGESHI.COM | N/A |
| Adobe Illustrator AI | Adobe | N/A |
| GraphPad Prism 9.5 | GraphPad Software Inc. | N/A |

**Table S3. Detailed longevity of flies with pan-neuronally knockdown of TBPH in two lines (VDRC 38377 and BDSC 39014) under the control of elav-GAL4**

| GAL4-system | elav-GAL4> | | | | | |
|---|---|---|---|---|---|---|
| Gender | Male | | | Female | | |
| Genotype | W- | TBPH RNAi (#38377) | TBPH RNAi (#39014) | W- | TBPH RNAi (#38377) | TBPH RNAi (#39014) |
| Average lifespan (days) ±s.d. | 58.1 ±13.93 | 40.83 ±12.33 | 27.19 ±9.193 | 72.12 ±12.14 | 43.63 ±13.48 | 51.71 ±10.71 |
| Median lifespan (days) | 60.5 | 41.5 | 26.5 | 74 | 46 | 53.5 |
| Statistical analysis | W- vs. RNAi-38377: **** *P* <0.000001<br>W- vs. RNAi-39014: **** *P* <0.000001<br>RNAi-38377 vs. RNAi-39014: **** *P* <0.000001 | | | W- vs. RNAi-38377: **** *P* <0.000001<br>W- vs. RNAi-39014: **** *P* <0.000001<br>RNAi-38377 vs. RNAi-39014: **** *P* =0.000008 | | |

**Table S4. Detailed longevity of flies with Motor neuron-specific knockdown of TBPH in two lines (VDRC 38377 and BDSC 39014) under the control of D42-GAL4**

| GAL4-system | D42-GAL4> | | | | | |
|---|---|---|---|---|---|---|
| Gender | Male | | | Female | | |
| Genotype | W- | TBPH RNAi (#38377) | TBPH RNAi (#39014) | W- | TBPH RNAi (#38377) | TBPH RNAi (#39014) |
| Average lifespan (days) ±s.d. | 53.8 ±17.66 | 44.38 ±14.23 | 27.78 ±13.83 | 67.3 ±15.12 | 37.4 ±17.07 | 47.55 ±14.44 |
| Median lifespan (days) | 55.25 | 48 | 31 | 70.5 | 42.5 | 47 |
| Statistical analysis | W- vs. RNAi-38377: **** *P* =0.000005<br>W- vs. RNAi-39014: **** *P* <0.000001<br>RNAi-38377 vs. RNAi-39014: **** *P* <0.000001 | | | W- vs. RNAi-38377: **** *P* <0.000001<br>W- vs. RNAi-39014: **** *P* <0.000001<br>RNAi-38377 vs. RNAi-39014: **** *P* =0.000217 | | |

**Table S5. Statistical analysis of the effect of enoxacin treatment targeting DCR on TBPH KO-induced locomotor deficiency**

| Classification | Genotype | W $^{-}$ | | TBPH $^{+/-}$ | | TBPH $^{-/-}$ | |
|---|---|---|---|---|---|---|---|
| | Enoxacin | + | − | + | − | + | − |
| Forward | Mean (times) ±s.d. | 342.0 ±90.49 | 280.3 ±97.77 | 209.3 ±41.51 | 179.8 ±61 | 127.2 ±32.21 | 143.7 ±26.16 |
| | DMSO vs Enoxacin | **** $P$ <0.0001 | | | | | |
| | Post hoc test | $P$ =0.3235 | | $P$ >0.9999 | | $P$ >0.9999 | |
| Reverse | Mean (times) ±s.d. | 33 ±17.48 | 46.83 ±33.93 | 56.17 ±12.02 | 59.67 ±26.44 | 127.0 ±79.01 | 72.67 ±27.9 |
| | DMSO vs Enoxacin | ** $P$ =0.0026 | | | | | |
| | Post hoc test | $P$ >0.9999 | | $P$ >0.9999 | | $P$ =0.0704 | |
| Pause | Mean (times) ±s.d. | 145.0 ±32.02 | 182.2 ±51.1 | 141.7 ±31.17 | 163.0 ±34.6 | 176.5 ±60.04 | 172.2 ±74.09 |
| | DMSO vs Enoxacin | $P$ =0.5634 | | | | | |
| | Post hoc test | $P$ =0.6189 | | $P$ >0.9999 | | $P$ >0.9999 | |
| Head sweep | Mean (times) ±s.d. | 172.0 ±40.95 | 202.5 ±13.02 | 224.5 ±32.73 | 254.2 ±50.86 | 217.7 ±81.12 | 221.2 ±65.34 |
| | DMSO vs Enoxacin | $P$ =0.0627 | | | | | |
| | Post hoc test | $P$ =0.9591 | | $P$ =0.9987 | | $P$ >0.9999 | |
| Turn | Mean (times) ±s.d. | 208.0 ±83.29 | 188.2 ±83.95 | 268.3 ±36.64 | 243.3 ±38.49 | 251.7 ±83.39 | 290.3 ±107.9 |
| | DMSO vs Enoxacin | $P$ =0.0644 | | | | | |
| | Post hoc test | $P$ >0.9999 | | $P$ >0.9999 | | $P$ >0.9999 | |
